# Systematic Analysis of Blood Cell Transcriptome in End-Stage Chronic Respiratory Diseases

**DOI:** 10.1371/journal.pone.0109291

**Published:** 2014-10-20

**Authors:** Julie Chesné, Richard Danger, Karine Botturi, Martine Reynaud-Gaubert, Sacha Mussot, Marc Stern, Isabelle Danner-Boucher, Jean-François Mornex, Christophe Pison, Claire Dromer, Romain Kessler, Marcel Dahan, Olivier Brugière, Jérôme Le Pavec, Frédéric Perros, Marc Humbert, Carine Gomez, Sophie Brouard, Antoine Magnan, the COLT Consortium

**Affiliations:** 1 UMR_S 1087 CNRS UMR_6291, l′Institut du Thorax, Université de Nantes, CHU de Nantes, Centre National de Référence Mucoviscidose Nantes-Roscoff, Nantes, France; 2 Université de Nantes, Nantes, France; 3 Institut National de la Santé et de la Recherche Médicale INSERM U1064, and Institut de Transplantation Urologie Néphrologie du Centre Hospitalier Universitaire Hôtel Dieu, Nantes, France; 4 CHU de Marseille, Aix Marseille Université, Marseille, France; 5 Centre Chirurgical Marie Lannelongue, Service de Chirurgie Thoracique, Vasculaire et Transplantation Cardiopulmonaire, Le Plessis Robinson, France; 6 Hôpital Foch, Suresnes, France; 7 Université de Lyon, Lyon, France; 8 Université de Lyon 1, Lyon, France; 9 INRA, UMR_S 754, Lyon, France; 10 Hospices Civils de Lyon, Lyon, France; 11 Clinique Universitaire Pneumologie, CHU de Grenoble, Grenoble, France; 12 Université Joseph Fourier, Grenoble, France; 13 Inserm U1055, Grenoble, France; 14 European Institute of Systems Biology and Medicine, Lyon, France; 15 CHU de Bordeaux, Bordeaux, France; 16 CHU de Strasbourg, Strasbourg, France; 17 CHU de Toulouse, Toulouse, France; 18 Hôpital Bichat, Service de Pneumologie B et Transplantation Pulmonaire, Paris, France; 19 Université Paris-Sud, Le Kremlin-Bicêtre, France; 20 AP-HP, Service de Pneumologie, DHU Thorax Innovation, Hôpital Bicêtre, Le Kremlin-Bicêtre, France; 21 INSERM U999, LabEx LERMIT, Centre Chirurgical Marie Lannelongue, Le Plessis Robinson, France; Helmholtz Zentrum München/Ludwig-Maximilians-University Munich, Germany

## Abstract

**Background:**

End-stage chronic respiratory diseases (CRD) have systemic consequences, such as weight loss and susceptibility to infection. However the mechanisms of such dysfunctions are as yet poorly explained. We hypothesized that the genes putatively involved in these mechanisms would emerge from a systematic analysis of blood mRNA profiles from pre-transplant patients with cystic fibrosis (CF), pulmonary hypertension (PAH), and chronic obstructive pulmonary disease (COPD).

**Methods:**

Whole blood was first collected from 13 patients with PAH, 23 patients with CF, and 28 Healthy Controls (HC). Microarray results were validated by quantitative PCR on a second and independent group (7PAH, 9CF, and 11HC). Twelve pre-transplant COPD patients were added to validate the common signature shared by patients with CRD for all causes. To further clarify a role for hypoxia in the candidate gene dysregulation, peripheral blood mononuclear cells from HC were analysed for their mRNA profile under hypoxia.

**Results:**

Unsupervised hierarchical clustering allowed the identification of 3 gene signatures related to CRD. One was common to CF and PAH, another specific to CF, and the final one was specific to PAH. With the common signature, we validated T-Cell Factor 7 (*TCF-7*) and Interleukin 7 Receptor (*IL-7R*), two genes related to T lymphocyte activation, as being under-expressed. We showed a strong impact of the hypoxia on modulation of *TCF-7* and IL-7R expression in PBMCs from HC under hypoxia or PBMCs from CRD. In addition, we identified and validated genes upregulated in PAH or CF, including Lectin Galactoside-binding Soluble 3 and Toll Like Receptor 4, respectively.

**Conclusions:**

Systematic analysis of blood cell transcriptome in CRD patients identified common and specific signatures relevant to the systemic pathologies. *TCF-7* and *IL-7R* were downregulated whatever the cause of CRD and this could play a role in the higher susceptibility to infection of these patients.

## Introduction

Dependence on oxygen supplementation is an end-stage condition of several chronic respiratory diseases (CRD). In France, almost 150,000 patients receive long-term oxygen therapy, with a median survival of 1 to 4 years depending on the underlying cause [1]. Chronic Obstructive Pulmonary Disease (COPD), Cystic Fibrosis (CF) and Pulmonary Arterial Hypertension (PAH) have this end-stage supplementation in common despite distinct pathophysiologies and treatments. COPD results from damage to airways and lung parenchyma [2]; CF is caused by a mutation in the Cystic Fibrosis Transmembrane conductance Regulator gene (*CFTR*), affecting bronchial epithelium mucus production leading to lung impairment and infection [3]; PAH is a condition involving a remodelling of pulmonary vessels causing right heart failure [4], [5]. Chronic tissue hypoxia resulting from these diseases induces peripheral damage including weight loss and metabolism dysfunction directly impacting the patient outcome [6], [7]. Using high*-*throughput approaches in genomics, transcriptomics or proteomics, previous studies have identified biological signatures relevant for these diseases, characterized notably by immunological abnormalities [8]–[10]. However, the mechanisms of the systemic consequences of CRD are still poorly understood. Little attention has been paid to date to the impact of CRD on blood cells, which may carry disease-specific information due to direct or indirect modifications. We hypothesized that CRD-induced metabolic changes could impact blood cell gene expression, and that this compartment offers a means to detect genes implicated in unexplored CRD-related changes.

To test this hypothesis, a systematic analysis of blood mRNA profiles was performed in CRD patients awaiting lung transplant. Microarray analysis was performed on a first group (microarray cohort) composed of PAH, CF and Healthy Controls (HC). We distinguished a common transcriptomic signature related to CRD and specific signatures for each disease. These patterns were validated in an external and independent cohort of HC and patients with CF, PAH and COPD (validation cohort).

## Methods

### Study population

**Figure 1:**
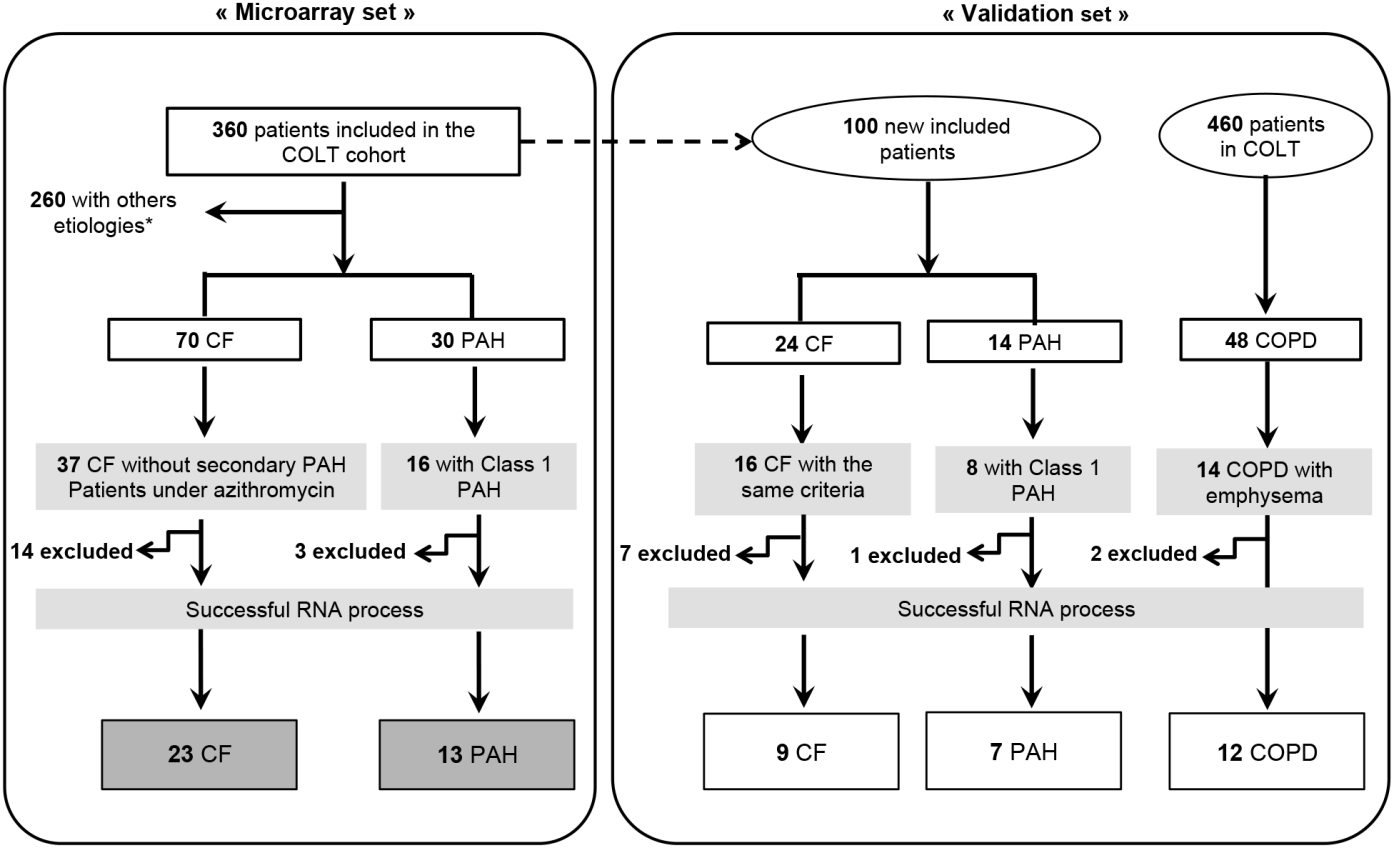
Strategy for selecting patients from the COLT (COhort of Lung Transplantation). *Emphysema, Sarcoïdosis, Lymphangiomatosis, Secondary PAH, histiocytosis, fibrosis, bronchiectasis and COPD (Chronic Obstructive Pulmonary Disease). CF: Cystic Fibrosis; PAH: Pulmonary Arterial hypertension; COPD: Chronic Obstructive Pulmonary Disease. Patients were excluded during the RNA process following specific qualities criteria.

The study protocol was approved by the Comité de Protection des Personnes Ouest 1-Tours (reference number: 2009-A00036-51), and written informed consent was obtained from all subjects. Blood samples were collected from pre-transplant patients included in a multicentric longitudinal cohort, intituled Cohort of Lung Transplantation (COLT). This cohort consists in monitoring patients during 5 years following lung transplantation in order to detect predictive factors of chronic lung allograft dysfunction. We took advantage of the biocollection to study blood of patients with CRD before transplantation. The strategy of selecting the included samples is described in Figure 1. To increase the chance of detecting a signature of CRD that is independent of the primary disease, we first focused on 2 diseases with highly contrasted pathophysiology, CF and Class 1 PAH. Secondarily, to validate this common signature as being present in any CRD, a supplementary group of COPD patients was included. Patients were selected among the cohort so that each group was as homogeneous as possible regarding the form of the disease (CF documented as exempt of secondary PAH, Class 1 PAH, COPD with documented emphysema) and treatment (CF with azithromycin). Some samples were secondarily excluded due to unsatisfying mRNA quality. The “microarray cohort” was therefore composed of 13 patients diagnosed with PAH and 23 with CF (Table 1A). Twenty-eight samples from healthy controls (HC) collected by the French Blood Establishment were used in the microarray analysis (Table 1A). In order to overcome the age difference between CF and PAH, we selected a HC population mixed in age: 46.43% of HC were matched with CF (born after 1970) and 53.57% with PAH patients (born before 1960).

**Table 1:** Summary of clinical data of patients included in the microarray (A) and in the validation (B) cohorts.

Patients Group		Age	Gender (Male/All)	Smoking status (Yes/No)	BMI (Kg/m2)	FEV1 (%)	PaO2 (kPa)	PAPm (mmHg)
A.	*Patients included in the microarray cohort*							
**CF n = 23**			11/23	0/23*				
	mean ± SD	24±7			18.4±2.25	24.7±8.50	8.1±0.74	
	min	16			14.5	14	7	
	max	41			22.4	42	9.6	
**PAH n = 13**			5/13	0/13^φ^				
	mean ± SD	41±15.3			22.3±6.10	74.9±30.05	7.9±1.66	66.7±17.17
	min	16			16.3	36	5.9	53
	max	61			34	122	10	110
**HC n = 28**			9/28					
	mean ± SD	42.5±14.20						
	min	21						
	max	68						
**B.**	***Patients included in the validation cohort***							
**CF n = 9**			5/9	0/9				
	mean ± SD	26±6.3			18±1.20	24±5.43	7.2±1.58	
	min	15			16	15	4.9	
	max	36			19	35	8.4	
**PAH n = 7**			0/7	0/7£				
	mean ± SD	34±18.5			24±7.81	80±15.06	9.1±0.22	64±23.39
	min	15			20	30	8.9	40
	max	67			27	95	11.2	100
**COPD n = 12**			8/12	0/12^§^				
	mean ± SD	58±2.6			21.2±4.53	18.1±5.40	7.84±1.9	
	min	54			16	10	3.8	
	max	61			28.6	32	10.17	
**HC n = 11**			6/11					
	mean ± SD	41.5±13.47						
	min	23						
	max	58						

PAH = pulmonary arterial hypertension; CF = cystic fibrosis; HC = healthy controls; COPD = chronic obstructive pulmonary disease; BMI = Body Mass Index; FEV1 = Forced Expiratory Volume in 1 second; PaO2 = arterial oxygen tension; PAPm = mean pulmonary artery pressure (mmHg);

*2/23 are wean smokers; ^φ^2/13 are wean smokers; ^£^1/7 is a wean smoker; ^§^12/12 are wean smokers.

The relevance of candidate genes from the microarrays analysis was confirmed by quantitative polymerase chain reaction (q-PCR) performed on a second group of patients referred to as the “validation cohort”. This group was composed of 7 PAH and 9 CF patients newly included in COLT since the first selection, 12 COPD patients selected among the whole COLT population, and 11 HC (Table 1B). The same criteria were applied as in the “microarray” set. In this validation cohort again, we matched HC according to the patients age: 54.54% were born before 1960 and 45.46% after 1970.

### RNA Isolation

Samples were collected in PAXgene tubes (PreAnalytix, Qiagen), and stored at −80°C. Total RNA was extracted using the PAXgene Blood RNA System kit with an on-column DNase digestion protocol. Quality and quantity of total RNA were determined using a 2100 Bioanalyzer (Agilent Technologies Incorporation) and a NanoDrop ND-1000 spectrophotometer (NanoDrop Technologies). Microarray and qPCR analyses were performed on RNA with 260/280 and 260/230 OD ratios above 1.8 and a RNA integrity number (RIN) above 7.

### Gene expression microarray analysis

RNA samples were prepared and hybridized on Agilent Human Gene Expression 8×60 K microarrays (Agilent Technologies, part number: G4851A). In order to avoid any bias due to blood populations differences between groups, the Lowess (locally weighted scatterplot smoothing) normalization procedure was applied on all the microarrays together [11]. Thereby spots with half of the samples exhibiting signals less than the mean of all median signals were removed (threshold: 90.83). 30,146 probes were kept out of 58,717. Raw microarray data were deposited in the Gene Expression Omnibus (GEO) database (accession number GSE38267). Unsupervised hierarchical clustering was performed with Cluster (v3.0) and TreeView software using uncentred Pearson correlation with a median-centred gene dataset. To select the genes participating to the same biological process, we selected 5 clusters (A–E), based on a combined approach: selected genes were clustered together and exhibited a between-group t-test p-value below 1% [12]. The biological significance for each cluster was determined using GOminer software. Thus over-represented GO ontology (GO) categories within the list of genes were identified by comparison with the others genes expressed on the microarrays (*i.e.* the 17,163 expressed genes). In addition, Ingenuity Pathway Analysis (IPA) (Ingenuity Systems Inc.) was used to construct network pathways.

### Sample classification methods from gene expression data

Prediction Analysis of Microarray (PAM) was performed using R v2.13.0 software with a *pamr* package to identify minimum gene sets that differentiated patient groups. Additional hierarchical clustering was performed with MultiExperiment Viewer software [13] using the uncentered Pearson correlation as a similarity metric and average linkage clustering. Principal component analysis (PCA) and receiver operating curve (ROC) were performed using R v2.13.0 software with *ade4* [14] or pROC package, respectively.

### Quantitative PCR (qPCR) for microarray validation

Microarray results were validated by qPCR with a new set of independent samples. Complementary DNA was synthesized starting from 500 ng of RNA using an Omniscript kit (Qiagen). Real-time quantitative PCR was performed on a ViiA7 Fast Real-Time PCR System (Applied Biosystems) using commercially available primers: *HPRT1* (Hs99999909_m1), β*2M* (Hs00984230_m1), *ACTB* (Hs99999903_m1), *TCF-7* (Hs00175273_m1), *CD6* (Hs00198752_m1), *IL-7R* (Hs00233682_m1), *LGALS3* (Hs00173587_m1), *MDK* (Hs00171064_m1) *TLR4* (Hs00152939_m1), *NLRC4* (Hs00802666_m1) and *TLR8* (Hs00607866_mH). Samples were analysed in duplicate and the geometric mean of quantification cycle values (Cq) for *HPRT1*, *β2M* and *ACTB* was used to normalize cDNA amounts. Relative expression between a sample and a reference was calculated according to the 2^−ΔΔCq^ method [15].

### Cellular culture under hypoxic and normoxic conditions

PBMCs from HC cultured in 24-well plates with 1 mL of RPMI 1640 media supplemented 10% FBS, 200 mg/mL penicillin, 200 U/mL streptomycin, 4 mM L-glutamine were placed either in a hypoxia incubator, created by displacing O_2_ (2% O_2_) with infusion of N_2_ (93%), or a normoxic incubator (21% O_2_) for 12 h at 37°C. RNA was extracted using a Macherey Nagel kit according to the supplier’s recommendations. Complementary DNA was synthetized from 250 ng using a superscript III kit (invitrogen) and qPCR was performed to study *TCF-7* and *IL-7R* expression. Finally, median fluorescence intensity was measured for CD127 (also called IL-7R) protein on CD3^+^CD4^+^ T cells by flow cytometry using the following antibodies (1/100e): CD3-PE-Cy7, CD4-PercP-Cy 5.5 and CD127-PE (BD, Biosciences). A Viability dye (BD Horizon V450, 1/1000^e^) may be used to exclude dead cells from analysis (LSR II BD Biosciences and FlowJo software).

### Analysis of lymphocyte flow cytometry profile in PBMCs of patients in CRDs

PBMC from 7 CF, 8 PAH and 6 COPD patients and 6 HC were rapidly thawed by placing cryovials at 37°C. Cells were washed, resuspended in supplemented RPMI 1640. 3.10^6^ cells were stained with CD3-PE-Cy7, CD4-PercP-Cy 5.5, CD127-PE, BD Horizon V450 (BD, Biosciences). Results were generated by flow cytometry (LSR II BD Biosciences and FlowJo software).

### Statistics

Regarding microarray analysis, the selection of genes of interest is based on a combined approach including a t-test with a p-value inferior to 0.01 and a clustering selection. This approach is based on the assumption that genes participating to same biological functions are clustered together as demonstrated by Alizadeh *et al.* [16]. A test called SP Calc was used for calculating sample size and power in our microarray study [17]. Our analysis exhibited a reasonable statistical power superior to 75% despite the small sample size. qPCR and Flow cytometry results are given as mean ±standard error of the mean. The non-parametric Kruskal Wallis tests with Dunn’s ad-hoc pairwise comparisons and Mann and Whitney test were applied using GraphPad Prism, v4. Differences between groups were defined as significant when the p-value was <0.05.

## Results

### Clinical Parameters

Table 1A shows the characteristics of the patients whose blood samples were used for the microarray process. The mean ages of PAH patients and HC were similar (41±15.3 years *vs* 42.5±14.20, mean ± SD, p≥0.05). However, CF patients were significantly younger than HC and PAH (24±7 *vs* 42.5±14.20 for HC, p≤0.01, and 41±15.3 for PAH, p≤0.05). In contrast, CF and PAH were comparable in mean body mass index (18.4±2.25 *vs* 22.3±6.10) and PaO2 (8.1±0.74 and 7.9±1.66 kPa) measured without supplementary oxygen. PAH patients were selected in line with the Group 1 of the Pulmonary Hypertension World Health Organisation (WHO) clinical classification system and displayed a mean pulmonary artery pressure (mPAP) of 66.7±17.17 mmHg. Table 1B gives the characteristics of the subjects whose blood samples served to confirm the microarray data by qPCR (validation cohort). Age of CF, PAH, COPD and HC did not significantly differ (26±6.3 (CF), 34±18.5 (PAH), 58±2.6 (COPD) and 41.5±13.47 (HC)). The means of PaO2 values did not differ significantly between groups of patients. Finally, Figure S1 shows significant differences in the microarray cohort between CF and PAH concerning total count (in Giga/L) of leukocytes, neutrophils and eosinophils (Figure S1A). However, these results do not influence the microarray analysis, normalized on the number of blood cells among each population. Noteworthy, no significant difference was found regarding proportions of leukocyte subpopulations between CF and PAH (Figure S1B). We observed no modification across all blood populations between COPD, PAH and CF in the validation cohort (Figure S1C, D).

### Overall gene expression profiles

**Figure 2:**
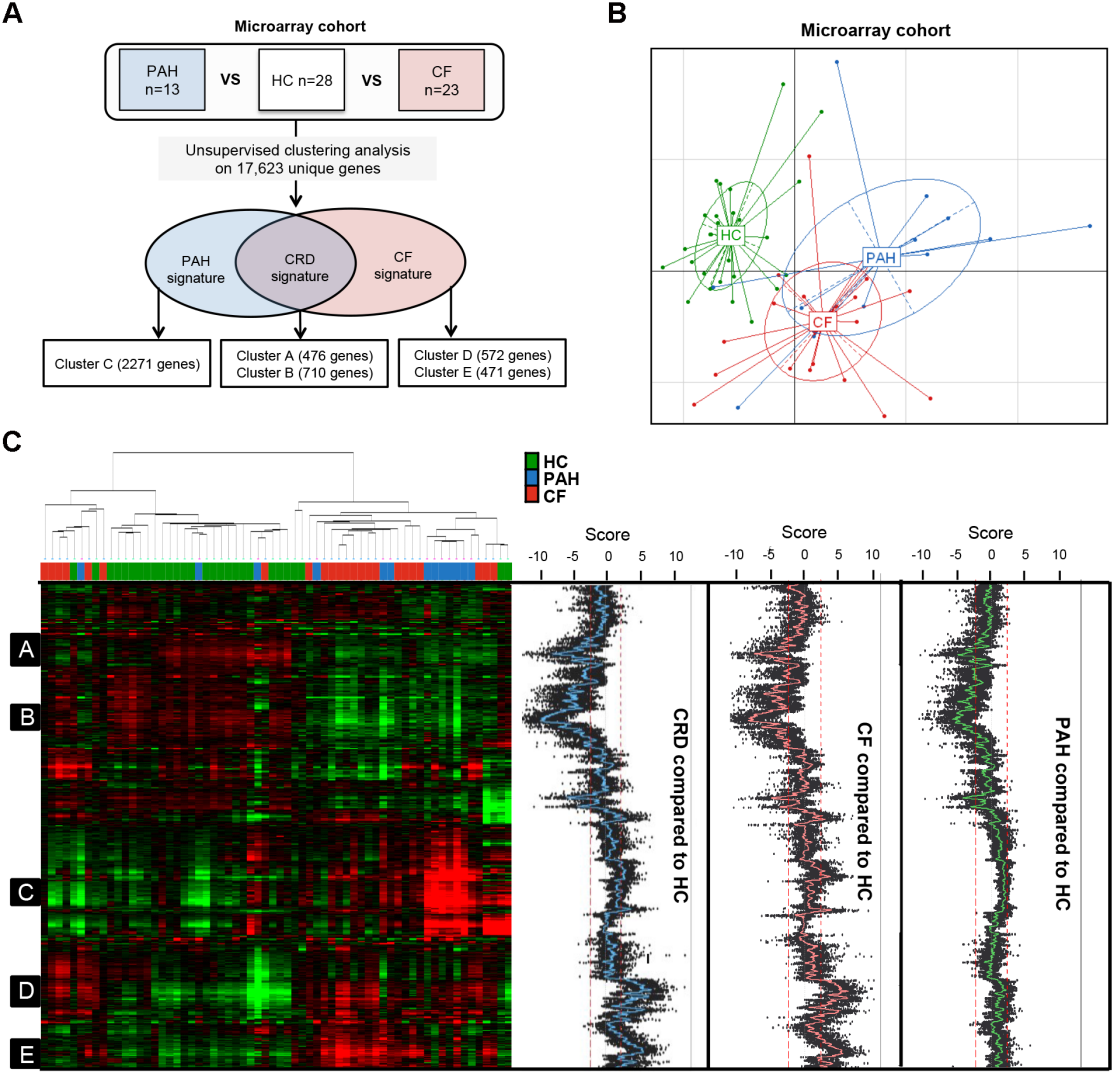
Unsupervised hierarchical clustering analysis. A) Tree analysis. Clustering analysis based on the 30,146 probes corresponding to 17,163 unique genes expressed in PAH, CF patients and Healthy Controls (HC). 3 signatures were found: 1 common between CF and PAH (named CRD signature), 1 specific to CF and 1 to PAH; B) Principal Component Analysis (PCA) displayed a clear separation between HC and patients with CRD, whereas CF and PAH patients were less distinct; C) 5 groups of genes (or clusters) were selected, A to E, based on a combined approach: selected genes were clustered together and exhibited a t-test p-value below 1% between the CRD group (PAH+CF), CF or PAH versus HC. Green represents relatively low expression, and red indicates relatively high expression.

Gene expression microarrays were performed using total RNA from peripheral whole blood from 23 CF, 13 PAH and 28 HC (Figure 2A). Using the expression values of 17,163 unique genes, the principal component analysis (PCA) graph based on the first 2 components displayed a clear separation between HC and the CRD patients, whereas there was less distinction between CF and PAH (Figure 2B). In addition, a similar segregation was observed in the sample dendrogram of the unsupervised hierarchical clustering (Figure 2C). We then used a gene clustering approach to select signatures associated with CRD, CF and PAH, assuming that genes clustering together participate in a common function [18]. Based on unsupervised hierarchical clustering and an associated student t-test (p-value<0.01), we identified two clusters of under-expressed genes (clusters A (476 genes) and B (710 genes)) in both CF and PAH groups compared to HC (Figure 2A, C). In addition, compared to HC one cluster was associated with PAH (cluster C = 2,271 genes) and two with CF (clusters D and E, composed of 572 and 471 genes, respectively) (Figure 2A, C). These latter 3 clusters are composed of over-expressed genes relative to HC. In order to investigate the biological significance of these 5 clusters, GOminer analysis was performed to annotate all genes in each cluster. The ingenuity pathway analysis was used to identify key functional pathways.

### Identification and validation of genes associated with both CRDs

**Figure 3:**
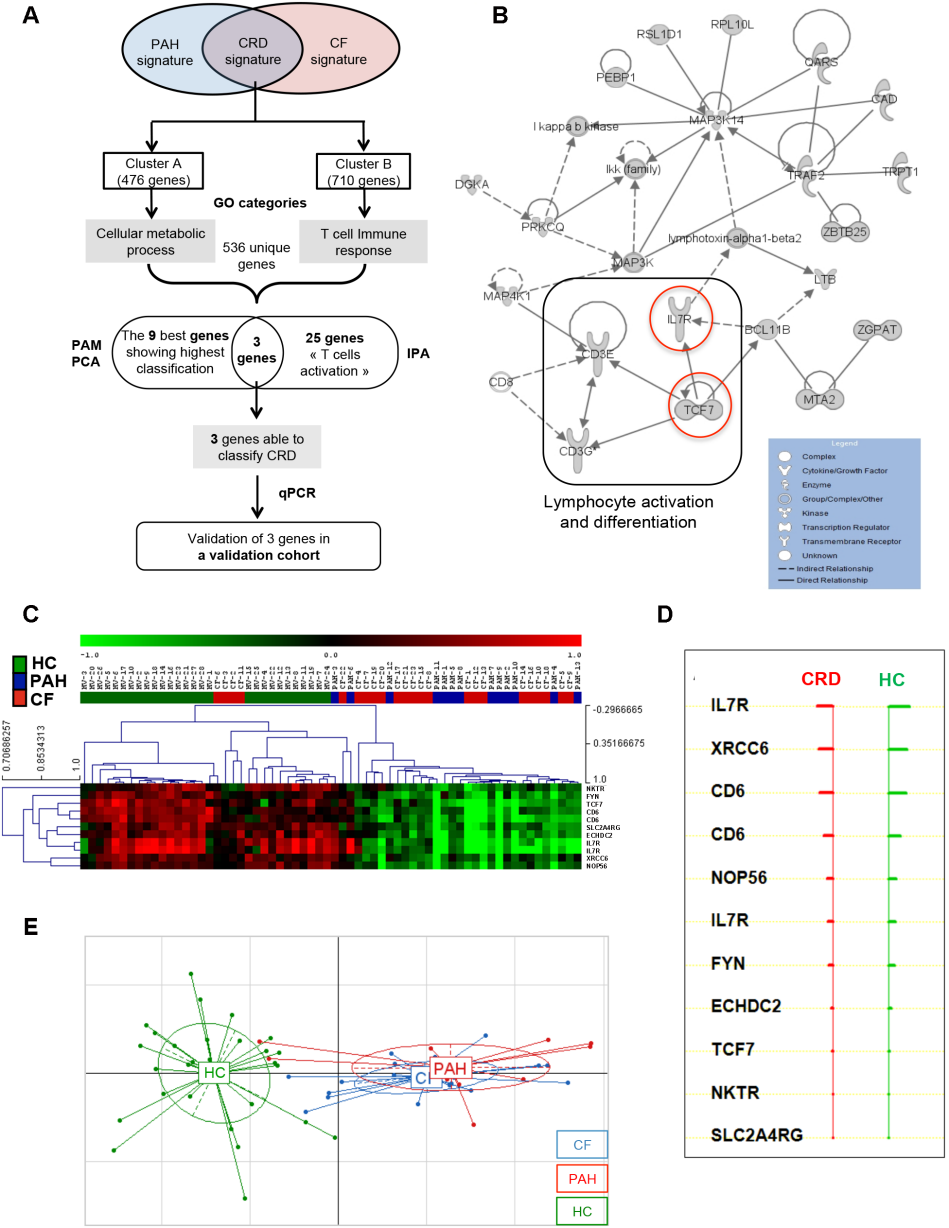
Characterization of under-expressed genes in the CRD signature. A) Tree analysis of the CRD signature. Identification of the most representative genes by Gominer, PAM, IPA analysis and validation of the candidate genes in the validation cohort by qPCR; B) Network generated by IPA on the most significant GO categories in the CRD signature. Solid lines indicate *direct* interactions and dashed lines represent *indirect* interactions. Under-expressed genes in CRD are in gray. C) PAM analysis based on the most representative GO categories of Cluster A and B. Green represents relatively low expression, and red indicates relatively high expression. D) List of 9 genes able to classify correctly CRD and HC. E) The PCA graph of the 9 genes identified by PAM analysis indicated a clear separation between HC and patients with CRD (CF and PAH).

Microarray data highlighted two under-expressed genes signatures in both diseases (Figure 3A). Cluster A was mainly related to cellular metabolic processes (GO:0044237) (Table 2A) including genes involved in the cell cycle (cyclin-dependent kinase 9 (*CDK9*), ataxia telangiectasia mutated (*ATM*) and B-cell CLL/lymphoma 2 (*BCL2*)). Concerning cluster B, we found GO categories related to T cell signalling (such as “T cell receptor signalling pathway”, GO:0050852 and “antigen receptor-mediated signalling pathway”, GO:0050851) (Table 2B). Among genes from cluster B (associated with CRD) and from enriched GO categories (mainly related to gene expression and T cell signalling), we identified a main network of 25 genes associated with lymphocyte survival including 4 major genes under-expressed in both CRDs: CD3 gamma (*CD3G*), CD3 Epsilon (*CD3E*), Transcription factor 7 (*TCF-7*) and Interleukin-7 Receptor (*IL-7R*) (Figure 3B).

**Table 2:** Gene Ontology (GO categories) for genes in CRD signature versus HC.

GO ID	GO category	Number of significant genes	Enrichment	FDR
A.	*Cluster A - CRD signature*			
GO:0044260	cellular macromolecule metabolic process	165	1.39	0.00
GO:0043170	macromolecule metabolic process	169	1.32	0.00
GO:0090304	nucleic acid metabolic process	117	1.49	0.00
GO:0006139	nucleobase nucleoside nucleotide and nucleic acid metabolic process	128	1.42	0.00
GO:0044237	cellular metabolic process	189	1.23	0.00
GO:0044238	primary metabolic process	188	1.23	0.00
GO:0034641	cellular nitrogen compound metabolic process	132	1.36	0.00
GO:0006807	nitrogen compound metabolic process	133	1.34	0.00
GO:0008152	metabolic process	199	1.17	0.00
GO:0010467	gene expression	108	1.38	0.00
GO:0016070	RNA metabolic process	81	1.48	0.01
GO:0010556	regulation of macromolecule biosynthetic process	83	1.46	0.01
GO:0031323	regulation of cellular metabolic process	101	1.37	0.01
GO:0031326	regulation of cellular biosynthetic process	84	1.42	0.01
GO:2000112	regulation of cellular macromolecule biosynthetic process	80	1.44	0.02
GO:0019222	regulation of metabolic process	107	1.33	0.01
**B.**	***Cluster B - CRD signature***			
GO:0006414	translational elongation	26	4.93	0.00
GO:0006415	translational termination	23	4.74	0.00
GO:0031018	endocrine pancreas development	23	4.69	0.00
GO:0031016	pancreas development	23	4.45	0.00
GO:0035270	endocrine system development	23	3.80	0.00
GO:0006364	rRNA processing	18	3.79	0.00
GO:0016072	rRNA metabolic process	18	3.67	0.00
GO:0043624	cellular protein complex disassembly	24	3.56	0.00
GO:0008033	tRNA processing	12	3.54	0.01
GO:0006399	tRNA metabolic process	19	3.53	0.00
GO:0019080	viral genome expression	24	3.51	0.00
GO:0019083	viral transcription	24	3.51	0.00
GO:0043241	protein complex disassembly	24	3.48	0.00
GO:0034470	ncRNA processing	30	3.44	0.00
GO:0050852	T cell receptor signaling pathway	14	3.39	0.00
GO:0034623	cellular macromolecular complex disassembly	24	3.36	0.00
GO:0042254	ribosome biogenesis	21	3.32	0.00
GO:0032984	macromolecular complex disassembly	24	3.28	0.00
GO:0034660	ncRNA metabolic process	39	3.23	0.00
GO:0022613	ribonucleoprotein complex biogenesis	28	3.00	0.00
GO:0050851	antigen receptor-mediated signaling pathway	15	2.93	0.01
GO:0006412	translation	56	2.88	0.00
GO:0071843	cellular component biogenesis at cellular level	28	2.88	0.00
GO:0019058	viral infectious cycle	27	2.84	0.00
GO:0071845	cellular component disassembly at cellular level	27	2.74	0.00
GO:0002429	immune response-activating cell surface receptor signaling pathway	15	2.74	0.01
GO:0022411	cellular component disassembly	27	2.69	0.00
GO:0022415	viral reproductive process	27	2.61	0.00
GO:0048610	cellular process involved in reproduction	31	2.58	0.00
GO:0006396	RNA processing	62	2.32	0.00

Only Gene Ontology (GO) categories enriched in cluster A (A) and in cluster B (B) with False Discovery rate (FDR) less than 1% and with more than 10 genes are displayed.

**Figure 4:**
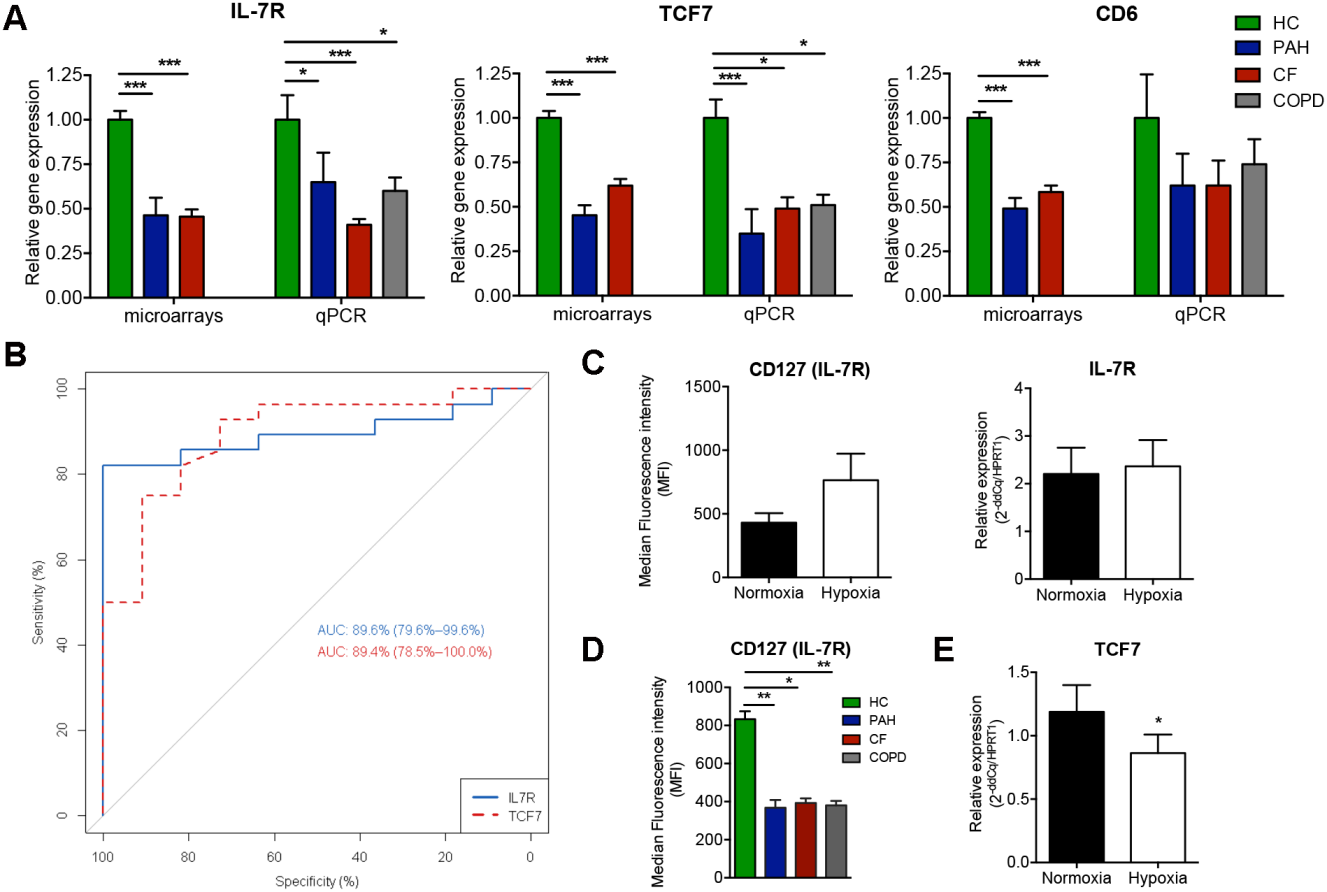
Validation of the most representative genes in the validation cohort. A) Quantitative PCR validation of 3 genes from the PAM analysis (*IL-7R*, *TCF-7* and *CD6*) using the validation cohort; B) based on these qPCR values, *IL-7R* and *TCF-7* enabled good discrimination between patients with CRD and HC, according to a receiver operating characteristic (ROC) analysis (AUC = 89.6% with p<0.001 and AUC = 89.4% with p<0.001, respectively); C) Median intensity fluorescence (MFI) and mRNA expression of *IL-7R* in PBMCs from healthy controls (HC) cultivated 12 hours under hypoxic and normoxic condition; D) MFI of IL-7R on PBMCs from CRD patients compared to HC; E) *TCF-7* expression in PBMCs from HC under hypoxia or normoxia.

We performed a Prediction Analysis of Microarrays (PAM) based on genes from enriched GO categories for clusters A and B (536 unique genes) in order to define the genes specifically modulated in CRD (Figure 3C). A combination of 11 probes corresponding to 9 unique genes successfully classified CRD patients, with only 4 out of 28 HC misclassified (PAM overall error = 14%) (Figure 3D). PCA analysis based on the expression of these 9 genes clearly separated CRD from HC, suggesting a strong involvement of these genes in both CRDs (Figure 3E). Based on PAM and IPA analysis, we focused on *CD6*, *IL-7R* and *TCF-7*, 3 genes involved in lymphocyte activation. Transcript levels of these 3 genes were measured by qPCR in the validation cohort of 9 CF, 7 PAH, and 11 HC. To confirm the link between these genes and CRD, regardless of the primary disease, blood samples from 12 COPD patients were also analysed. We confirmed the under-expression of *TCF-7* and *IL-7R* in the three CRD groups expect for *CD6* (Figure 4A). The ROC analysis indicated that *IL-7R* and *TCF-7* discriminated CRD with high sensitivity and specificity (AUC = 89.6%; p<0.001 and AUC = 89.4%; p<0.001, respectively) (Figure 4B).

### Effect of hypoxia on TCF-7 and IL-7R expression

Finally, we investigated whether hypoxia itself, a hallmark of peripheral tissues in all end-stage CRD patients, regulates *TCF-7* and *IL-7R* genes. For this, we studied variation in the fluorescence intensity of IL-7R and expression of *TCF-7* and *IL-7R* in PBMCs from HC incubated 12h under hypoxic or normoxic conditions. No difference in median fluorescence intensity (MFI) or expression of IL-7R was observed, supposing a long-term action of hypoxia on its regulation (Figure 4C). However IL-7R was significantly downregulated in PBMC of CRD patients compared to HC (Figure 4D). Similarly, a significant decrease in *TCF-7* expression was found under hypoxia (Figure 4E). These results might suggest a possible modulation of *TCF-7* gene and IL-7R protein in response to the hypoxic state of patients with CRD.

### Identification and validation of genes associated with PAH

**Figure 5:**
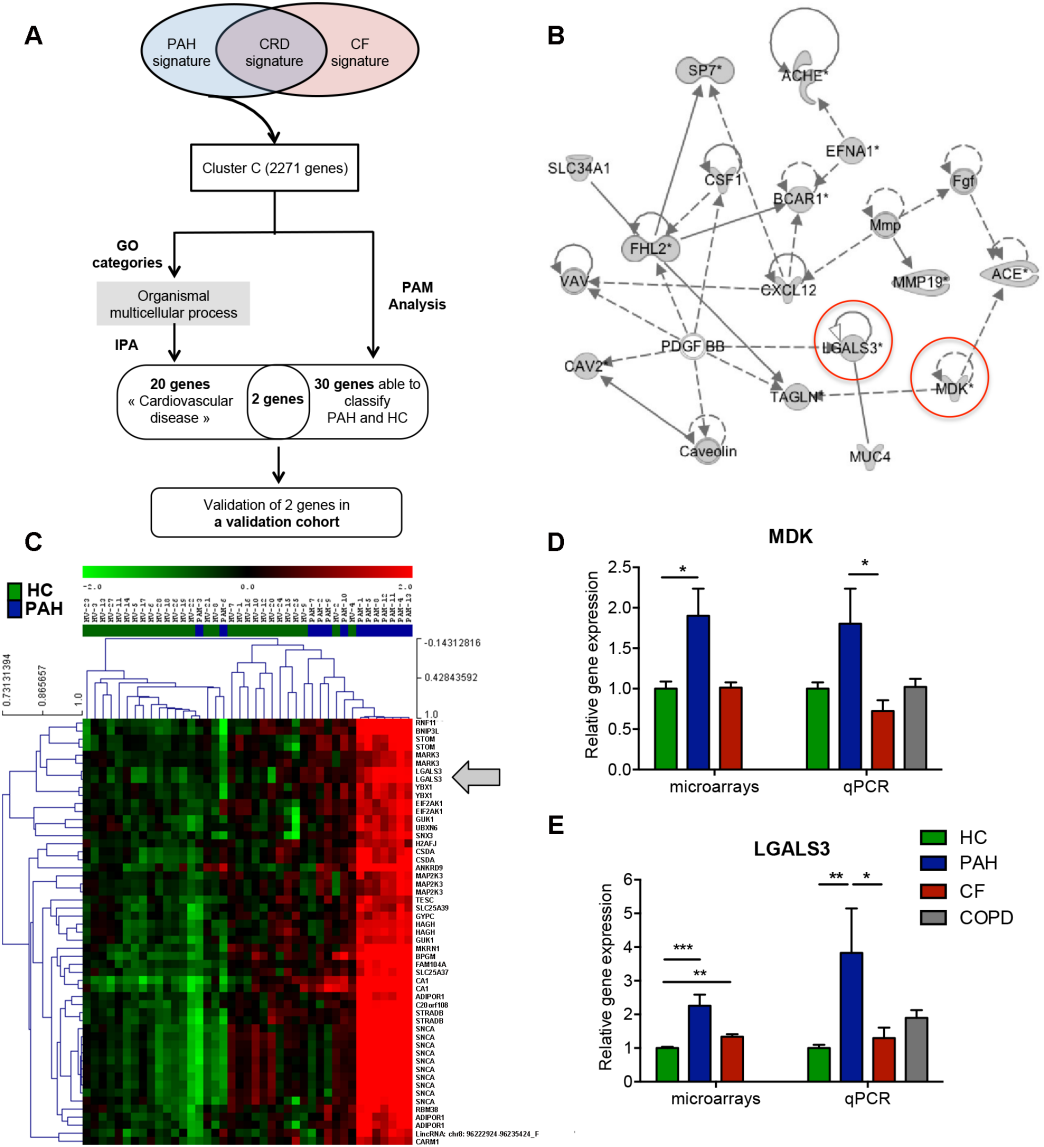
Characterization of over-expressed genes in the PAH signature. A) Tree analysis of the PAH signature. Identification of the most representative genes by Gominer, PAM, IPA analysis and validation by qPCR of the candidate genes in the validation cohort; B) Network generated by IPA on the most significant GO categories in PAH signature. Over-expressed genes in PAH are in gray. C) PAM analysis based on the cluster, *Green* represents relatively low expression, and *red* indicates relatively high expression; D and E) Validation by qPCR of *MDK* and *LGALS3* in the validation cohort, respectively.

Microarray analysis highlighted one cluster (cluster C) associated with PAH (Figure 5A). GO ontology analysis allowed us to identify genes related to “organismal multicellular process” (GO:0032501), “G-protein coupled receptor protein signaling pathway” (GO:0007186) and “sensory perception of smell” (GO:0007608) (Table 3). Using IPA analysis, we characterized several over-expressed genes in PAH, in particular related to cardiovascular diseases, including the genes coding for caveolin-2 (*CAV2*), the vasoconstrictor angiotensin-converting enzyme gene (*ACE*), as well as molecules involved in angiogenesis and adhesion, such as midkine (*MDK*), and lectin galactoside-binding soluble 3 (*LGALS3*) (Figure 5B). Similarly, using a PAM analysis based on the 2,271 genes for cluster C, we identified the most informative genes in the PAH-specific signature. A combination of 30 unique genes (corresponding to 53 probes) classified PAH accurately, with only 1 out of 28 HC misclassified (Figure 5C). Based on PAM and IPA analysis, we validated by qPCR two genes: *MDK* and *LGALS3* (Figure 5D, E). *MDK* was clearly over-expressed in PAH compared to the 3 other groups, but difference was only significant with CF (p<0.05) (Figure 5D). *LGALS3* was significantly over-expressed in PAH compared to HC and CF (p<0.01 and p<0.05, respectively) implying a strong contribution of this gene to PAH (Figure 5E).

**Table 3:** Gominer Analysis based for over-expressed genes in PAH signature versus HC.

GO ID	GO category	Number of significant genes	Enrichment	FDR
GO:0032501	multicellular organismal process	323	1.29	0.00
GO:0007608	sensory perception of smell	18	4.28	0.00
GO:0003008	system process	104	1.65	0.00
GO:0007275	multicellular organismal development	243	1.33	0.00
GO:0007606	sensory perception of chemical stimulus	20	3.40	0.00
GO:0032502	developmental process	259	1.28	0.00
GO:0007186	G-protein coupled receptor protein signaling pathway	57	1.85	0.00
GO:0048731	system development	201	1.33	0.00
GO:0048856	anatomical structure development	219	1.30	0.00
GO:0050877	neurological system process	74	1.65	0.00
GO:0009187	cyclic nucleotide metabolic process	21	2.61	0.00
GO:0009190	cyclic nucleotide biosynthetic process	19	2.75	0.01
GO:0048513	organ development	141	1.36	0.01
GO:0030154	cell differentiation	150	1.34	0.01
GO:0030218	erythrocyte differentiation	16	2.90	0.01
GO:0031279	regulation of cyclase activity	14	3.12	0.01
GO:0030802	regulation of cyclic nucleotide biosynthetic process	16	2.85	0.01
GO:0030808	regulation of nucleotide biosynthetic process	16	2.85	0.01
GO:0007155	cell adhesion	65	1.58	0.01

Only GO categories enriched in cluster C with FDR less than 1%, with enrichment superior to 2 and with more than 10 genes are displayed.

### Identification and validation of genes associated with CF

**Figure 6:**
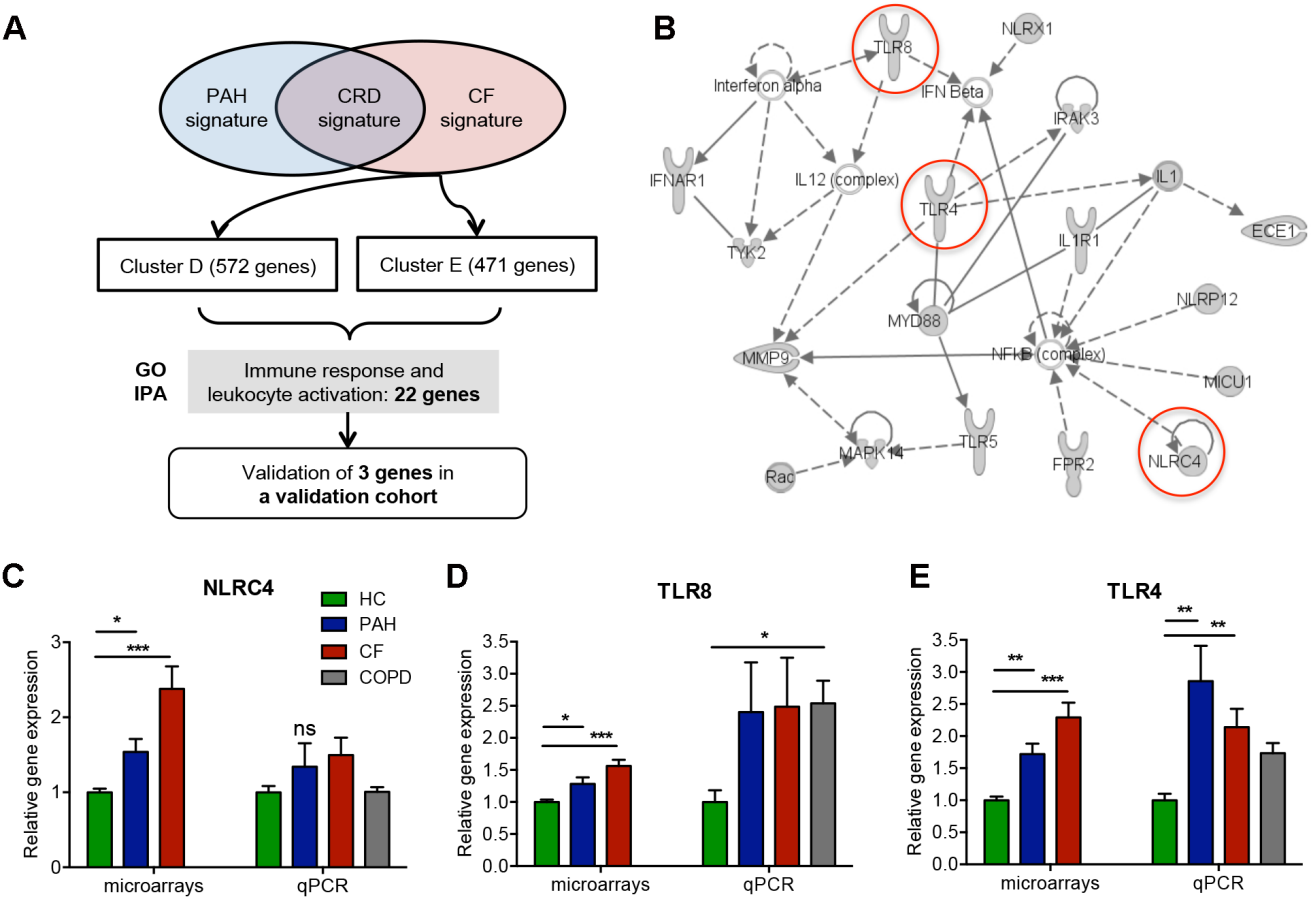
Characterization of over-expressed genes in the CF signature. A) Tree analysis of CF signature. Identification of the most representative genes by Gominer and IPA analysis and validation by qPCR of these genes in the validation cohort; B) Network generated by IPA on the most significant GO categories in CF signature. Over-expressed genes in CF are in gray; C, D, E) qPCR validation on the new cohort for *NLRC4*, *TLR8* and *TLR4* genes.

The CF signature was composed of two clusters (clusters D and E) of over-expressed genes involved in “cellular localization” (GO:0051641) (Table 4A) and in “Immune Response” (GO:0006955) and more specifically in “leukocyte activation” (GO:0002366) (Table 4B, Figure 6A). In addition, we found a set of 22 over-expressed genes associated with innate immunity, especially genes coding toll-like receptor 4 (*TLR4*), *TLR8*, NLR family CARD domain-containing protein 4 (*NLRC4*) and interleukin 1 (*IL1*) (Figure 6B). Using IPA, we measured the level of gene transcripts involved in CF, notably *NLRC4*, *TLR4* and *TLR8,* in the independent validation cohort. Whereas the over-expression of *NLRC4* and *TLR8* were not confirmed (Figure 6C, D), we found a significant increase in *TLR4* gene expression in CF patients (p<0.01 vs HC) (Figure 6E). Interestingly our investigation showed a significant expression of *TLR8* in COPD (p<0.05 vs HC) and *TLR4* in PAH, suggesting that these genes are not specific to CF. Altogether, these results still confirm the stimulation of innate immune response during CF.

**Table 4:** Gominer Analysis based for over-expressed genes in CF signature versus HC.

GO ID	GO category	Number of significant genes	Enrichment	FDR
A.	*Cluster D - CF signature*			
GO:0008104	protein localization	73	1.74	0.00
GO:0033036	macromolecule localization	82	1.63	0.00
GO:0045184	establishment of protein localization	63	1.73	0.01
GO:0016050	vesicle organization	12	4.46	0.01
GO:0015031	protein transport	62	1.73	0.00
GO:0006464	protein modification process	97	1.49	0.01
GO:0046907	intracellular transport	56	1.75	0.01
GO:0051641	cellular localization	77	1.57	0.01
GO:0034613	cellular protein localization	41	1.94	0.01
GO:0070727	cellular macromolecule localization	41	1.93	0.01
GO:0043412	macromolecule modification	99	1.45	0.01
GO:0016192	vesicle-mediated transport	49	1.78	0.01
GO:0044260	cellular macromolecule metabolic process	242	1.19	0.01
GO:0006886	intracellular protein transport	35	1.92	0.01
**B.**	***Cluster E - CF signature***			
GO:0009611	response to wounding	54	2.48	0.00
GO:0006952	defense response	53	2.45	0.00
GO:0023052	signaling	148	1.54	0.00
GO:0023033	signaling pathway	121	1.63	0.00
GO:0023046	signaling process	112	1.60	0.00
GO:0023060	signal transmission	111	1.58	0.00
GO:0007165	signal transduction	101	1.62	0.00
GO:0006955	immune response	51	2.13	0.00
GO:0002376	immune system process	70	1.84	0.00
GO:0048583	regulation of response to stimulus	47	2.17	0.00
GO:0035466	regulation of signaling pathway	60	1.94	0.00
GO:0035556	intracellular signal transduction	73	1.78	0.00
GO:0007243	intracellular protein kinase cascade	41	2.28	0.00
GO:0023014	signal transmission via phosphorylation event	41	2.28	0.00
GO:0006954	inflammatory response	27	2.85	0.00
GO:0002263	cell activation involved in immune response	14	4.78	0.00
GO:0002366	leukocyte activation involved in immune response	14	4.78	0.00
GO:0006950	response to stress	100	1.57	0.00
GO:0007169	transmembrane receptor protein tyrosine kinase signaling pathway	36	2.37	0.00
GO:0007166	cell surface receptor linked signaling pathway	74	1.73	0.00
GO:0023034	intracellular signaling pathway	80	1.65	0.00
GO:0050817	coagulation	28	2.61	0.00

Only GO categories enriched in cluster D (A) and in cluster E (B) with FDR less than 1%, with enrichment superior to 2 and with more than 10 genes are displayed.

## Discussion

The objectives of this research were to discover genes potentially involved in the peripheral damages seen in end-stage CRD by performing a systematic analysis of the blood mRNA from CRD patients. To make sure that genes identified as related to CRD were independent of the aetiology, we included in the screening analysis 2 diseases completely distinct in their pathophysiology, CF and PAH. We added a third unrelated disease, COPD, in the validation analysis, again increasing the probability that any link found to CRD was independent of the underlying diseases. This strategy not only provided CRD-related signatures, but also allowed the identification of genes specific of CF and PAH, some of which had not been suspected previously. The relevance of these disease-specific, highly contrasted signatures was reinforced by the concomitant study of 3 diseases that allowed each of them to serve as a control for the others and eliminate non-specific genes. COLT, a lung transplant cohort of 360 patients at time of this study, offered a unique opportunity to select homogeneous groups in terms of age, sex, underlying care and treatment, thus increasing the chances of detecting reliable signatures.

First, based on a microarray analysis using a combined hierarchical clustering approach, we identified a signature for CRD. The GO terms analysis in cluster A and B highlighted large families of genes associated with “metabolic process” and “T cell receptor signalling pathway”, respectively. A set composed of under-expressed genes was determined, including genes involved in T-cell receptor (TCR) signalling, namely *CD3E*, *CD3G*, *IL-7R* and *TCF-7* (also known as *TCF-1*). *TCF-7* and *IL-7R* were identified in the gene network related to lymphocyte activation and were among the 9 genes selected in the PAM analysis. Their significant under-expression was confirmed by qPCR in independent CF and PAH groups and in individuals with COPD. Interestingly, these two genes are dependent on activation of the Wnt/β-catenin pathway and are pivotal in the control of T lymphocyte survival [19], [20]. IL-7R composed of 2 chains, the common γ chain and the α chain (or CD127), mediates signalling of IL-7. IL-7R neutralization delays post-depletional T cell recovery through both the suppression of thymopoiesis and the inhibition of T cell homeostatic proliferation [19]. As for *IL-7R*, a number of works have established *TCF-7* as a critical regulator necessary for the maintenance of normal T-cell development, but also for the induction of many components of T-cell identity [20], [21]. Among the genes induced by *TCF-7*, there are T-cell essential transcription factors such as *Gata3*, as well as components involved in the regulation of TCR such as *IL-7R* [22]–[24]. Recently *TCF-7* has been shown to induce Th2 and Th17 inflammation, supporting the hypothesis that dysfunction of this pathway at any stage of T cell differentiation could lead to immune deficiency [21]. In light of the above, the down-regulation of *IL-7R* and *TCF-7* genes implies decreased adaptive immunity in end-stage CRD patients and could indirectly explain some infections and complications. A recent study described a systemic gene expression profile in patients with COPD [25]. Among the candidate genes found, *TCF-7* was a biomarker for COPD. However, our study clearly shows that *TCF-7* is not specific of COPD, but is rather an important marker of CRD. Indeed *TCF-7* was down regulated in COPD, but also in CF and PAH, evidencing a modulation of *TCF-7* in response to respiratory failure, whatever its cause. Since functional immunity is maintained by the metabolic requirement of proteins [26], the alteration of the immune response in end-stage CRD could be related to under-nutrition.

Whether the signature is related to CRD whatever the stage, or is specific to advanced respiratory failure, should be elucidated in cohorts with less developed respiratory diseases. In addition, to make sure that the signature is specific to CRD, other chronic invalidating diseases involving under-nutrition should be investigated. However the direct hypoxia-driven down-regulation of *TCF-7* in PBMC suggests that it is a respiratory-related signature. The consequences of hypoxia were also confirmed by the down-modulation of IL-7R on the surface of PBMC from CRD patients. An improvement in the gene or protein expression after lung transplant would confirm the direct link with respiratory disorders.

In addition, we identified a specific signature for PAH. The GO and IPA analysis identified a number of genes already described in the pathophysiology of PAH: proteins coded by *CAV2* regulating lung endothelial cell proliferation and differentiation, and by *ACE*, a key enzyme in cardiovascular pathobiology, which serum levels are correlated with lung endothelial injury [27], [28]. Furthermore, the midkine (*MDK*) gene, a heparin-binding growth factor linked to *ACE*, was identified in our gene network. MDK is known to promote vascular leukocyte infiltration and migration and proliferation of smooth muscle cells. MDK levels are increased in systemic hypertension and *MDK* interacts with *ACE* in the renin-angiotensin system [29], [30].

The gene most contributing to PAH according to the PAM analysis was *LGALS3*, a member of the galectin family of carbohydrate binding proteins. We validated its over-expression in an independent PAH group by qPCR. Galectin-3 is described as a multifunctional protein involved in a variety of biological processes including fibrosis, angiogenesis and the activation of various immune cells, such as macrophages, neutrophils, mast cells and lymphocytes [31], [32]. Interestingly, several works have shown that the upregulation of *LGALS3* is linked to heart failure and is an independent blood biomarker for ventricular remodeling and mortality [33]–[35]. Our results suggest that *LGALS3* may be involved in right heart failure, the most common cause of death in PAH [36]. Further investigations are required to decipher the functional role of galectin-3 in PAH.

The over-expression of genes related to innate immunity identified in CF through GO analysis was consistent with inflammation in this disease [37]. In accord with this, the IPA analysis showed many genes involved in inflammatory functions. Among these, Pattern Recognition Receptor (PRR) family genes, including TLR, notably *TLR4* and *TLR8*, were overexpressed. Their altered expression is directly associated with immune-deregulation [38], [39]. We validated the *TLR4* over-expression by qPCR in blood from other CF patients. Most interestingly, *TLR4* was also significantly increased in uninfected PAH patients, suggesting that *TLR4* upregulation in CF patients is not related to infections. Additional PRRs were present in this network, including a member of the Nod-Like Receptor family, *NLRC4*. The *NLRC4* inflammasome is essential for host immunity against extracellular pathogens, such as *Pseudomonas aeruginosa*, a frequent pulmonary pathogen in CF [40]. Thus, *NLRC4* over-expression in the blood of CF could relate to their infectious status.

The number of patients per group is small, which can be seen as a limitation of the study. Indeed as showed on Figure 1 the selection strategy that aimed to get homogeneity of the patients populations within groups regarding type of the disease, treatments, experimental process (RNA and cDNA qualities) led to eliminating many samples from the analysis. The number of analysed patients was further decreased by elimination of unsuitable RNA. This strategy reduced the power of the study to detect genes relevant for each disease, but also lowered the risk of misclassifying the patients because of comorbidities. In addition, confounding factors such as age, lower in CF, or specific treatments, still cannot be eliminated. Nevertheless, we tried to overcome this by matching the age of HC population with these of CF and PAH groups. A systematic strategy of propensity score matching would have stratified group comparisons on such covariates. However the study was mainly designed to detect a signature common to CF and PAH that could be validated in new sets of patients and COPD, rather than identifying genes specific of each disease. A different strategy of selection aiming to eliminate confounding factors would have been applied if the discovery of genes specific for each disease had been the primary objective. Despite this limitation, clustering analysis discriminated groups accurately, provided functional clusters, and most significant genes were validated in independent samples. Moreover, the risk of bias is lowered by that overexpressed genes are related to pathophysiological pathways already known to be disturbed in the respective diseases. It is the case of innate immunity genes in CF.

The blood gene expression profiling of patients with CRD enabled us providing a systematic description of peripheral molecular events related to CRD, CF and PAH. Notably, a common pattern associated with respiratory diseases, mainly under-expressed genes playing a role in immune functions is described. The relevance of these genes in the immunodeficiency of CRD patients is potentially important and would benefit from being investigated in functional studies. Our study further demonstrates the interest of systematic gene screening in order to detect unexplored mechanisms. The peripheral signatures found strengthen the arguments for a global approach of respiratory diseases in a systemic medical strategy. Elucidation of the molecular mechanisms involved in these changes in gene expression will require further investigations.

## Supporting Information

Figure S1 **Blood cell count in Giga/L and in percentage in the microarray cohort (A and B) and the validation cohort (C and D).** Results are given as mean ± standard error (SEM). PAH = pulmonary arterial hypertension; CF = cystic fibrosis; COPD: Chronic Obstructive Respiratory Disease; Leu: Leukocytes; PMN: Polymorphonuclear Neutrophil; Ly: Lymphocytes; Mo: Monocytes; Eo: Eosinophils; Bas: Basophils.
(TIF)
