# Supplementary figures and images for: Systematic Analysis of Blood Cell Transcriptome in End-Stage Chronic Respiratory Diseases
Source: PLoS One. 2014 Oct 20;9(10):e109291. doi: 10.1371/journal.pone.0109291 (PMC4203719; doi:10.1371/journal.pone.0109291)

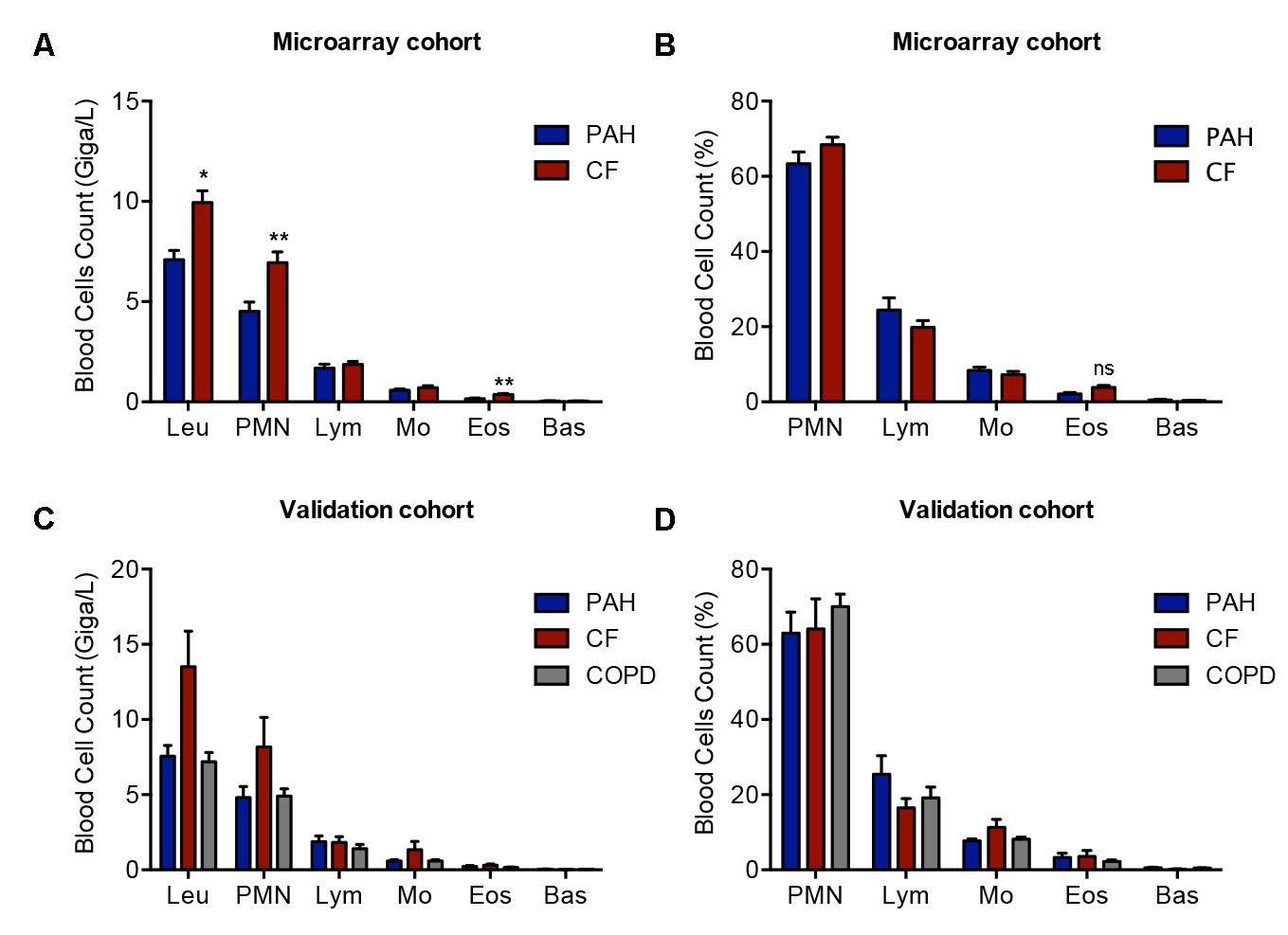

Supplement: Figure S1 — Blood cell count in Giga/L and in percentage in the microarray cohort (A and B) and the validation cohort (C and D). Results are given as mean ± standard error (SEM). PAH = pulmonary arterial hypertension; CF = cystic fibrosis; COPD: Chronic Obstructive Respiratory Disease; Leu: Leukocytes; PMN: Polymorphonuclear Neutrophil; Ly: Lymphocytes; Mo: Monocytes; Eo: Eosinophils; Bas: Basophils. (TIF) [file pone.0109291.s001.tif]
